# The majority of elite and professional athletes return to the preinjury level of activity after anterior cruciate ligament reconstruction: A systematic review and meta‐analysis

**DOI:** 10.1002/ksa.70020

**Published:** 2025-08-31

**Authors:** Riccardo D'Ambrosi, Andrea Marchetti, Luca Farinelli, Amit Meena, Piero Franco, Luca Maria Sconfienza, Riccardo Cristiani, Elmar Herbst, Christoph Kittl, Mirco Herbort, Elisabeth Abermann, Christian Fink

**Affiliations:** ^1^ IRCCS Ospedale Galeazzi – Sant'Ambrogio Milan Italy; ^2^ Department of Biomedical Sciences for Health University of Milan Milan Italy; ^3^ Department of Medicine, Surgery and Health Sciences, Orthopedics and Traumatology Unit University of Trieste Trieste Italy; ^4^ Clinical Orthopaedics Department of Clinical and Molecular Sciences Università Politecnica delle Marche Ancona Italy; ^5^ IRCCS INRCA Ancona Italy; ^6^ Department of Orthopaedics Shalby Hospital Jaipur India; ^7^ Department of Orthopedics Surgery, A.O.U. Careggi CTO University of Florence Florence Italy; ^8^ Department of Molecular Medicine and Surgery, Section of Sports Medicine Karolinska Institutet Stockholm Sweden; ^9^ Stockholm Sports Trauma Research Center (SSTRC) FIFA Medical Center of Excellence Stockholm Sweden; ^10^ Department of Trauma, Hand and Reconstructive Surgery University of Muenster Muenster Germany; ^11^ OCM Clinic Munich Steinerstraße Germany; ^12^ Gelenkpunkt‐Sports and Joint Surgery FIFA Medical Centre of Excellence Innsbruck Austria; ^13^ Research Unit for Orthopaedic Sports Medicine and Injury Prevention (OSMI) Private University for Health Sciences Medical Informatics and Technology Innsbruck Austria

**Keywords:** ACL, elite athletes, failure rate, return to sport, same preinjury level, time to return

## Abstract

**Purpose:**

To compare return to play (RTP), time to RTP, level of RTP, and anterior cruciate ligament (ACL) graft failure among elite and professional athletes from different sports after anterior cruciate ligament reconstruction (ACLR).

**Methods:**

The PubMed, Embase and Cochrane Library databases were searched to identify potentially relevant research articles that analysed RTP, time to RTP, level of RTP, and graft failure rate in elite and professional athletes after ACLR. An elite or professional athlete was defined as one who participates in national‐ or international‐level competitions in professional or amateur sports—including academy players aged 15 years or over.

**Results:**

A total of 49 studies met the inclusion criteria. Eleven different sports and 4463 knees were included in the final analysis. The pooled data revealed an RTP rate of 85.8% (95% confidence interval [CI] 82.8–88.5]. A lower RTP was observed in Australian football (67.8% [95% CI 54.1–80.1]; *p* < 0.001) and football (73.0% [95% CI 65.9–79.5]; *p* < 0.001) than in soccer (92.8% [95% CI 89.3–95.7]). Almost 90% of the athletes returned to their preinjury level. The meta‐analysis revealed no difference (*p* > 0.05) in the level of RTP rate among the different studies, ranging from 79.0% (soccer) to 97.3% (basketball). The pooled mean time to RTP was 292 days (95% CI 268–316 days). The pooled ACL graft failure rate was estimated to be 7.0% for athletes.

**Conclusions:**

Following ACLR, more than 85% of elite and professional athletes returned to play and almost 90% returned to their preinjury level, with a graft failure rate of 7.0% and a mean return to play at 292 days. Athletes and their treating physicians can utilise these findings to set reasonable expectations for their return to competition after ACLR.

**Level of Evidence:**

Level IV systematic review and meta‐analysis

**Abbreviations**
ACL: anterior cruciate ligament
ACLR: anterior cruciate ligament reconstruction
ALL: anterolateral ligament
BPTB: bone–patellar tendon–bone
CCTs: controlled (nonrandomized) clinical trials
CI: confidence interval
HT: hamstring tendon
MCL: Medial collateral ligament
MINORS: Methodological Index for Nonrandomized Studies score
NBA: National Basketball Association
NFL: National Football League
NHL: National Hockey League
PRISMA: Preferred Reporting Items for Systematic Reviews and Meta‐Analyses
RCTs: randomised controlled trials
REML: restricted maximum likelihood
RTP: return to play
SD: standard deviation

## INTRODUCTION

Anterior cruciate ligament (ACL) rupture is a serious and potentially career‐limiting injury for elite and professional athletes, particularly those involved in sports that include jumping, pivoting and cutting movements [14, 44, 60, 73, 75].

In most athletes, ACL reconstruction (ACLR) is performed to allow them to return to their preinjury level of sport [36, 40]. Despite advances in ACLR surgical techniques, rehabilitation protocols, and return‐to‐play (RTP) criteria, approximately 80% of ACL‐reconstructed patients return to some type of sports activity, but only 65% are able to return to their preinjury level and 55% return to competitive level sports [15, 32, 36]. In particular, sports involving jumping, pivoting, and cutting manoeuvres are associated with lower return to sport rates after ACL reconstruction [15, 32, 36].

Elite and professional athletes have higher RTP rates than the general population [15, 54, 81]. Differences between sports and their unique physical demands contribute to these variations. In fact, RTP rates range from 77% in Australian Football and Major League Soccer to 100% in alpine skiing [36, 63, 86].

However, the treatment of ACL injuries in elite and professional athletes presents the additional challenge of restoring their ability to perform at an exceptionally high physical level [15]. Sport‐specific demands are also reflected in performance after ACLR. National Hockey League (NHL) players have the highest RTP rates and shortest recovery times, with performance rates that do not differ from preinjury levels. National Football League (NFL) athletes have decreased athletic performance and the shortest postoperative careers [9, 18, 49]. National Basketball Association (NBA) players showed a decline in performance from preinjury levels, with an average career length of 4.3 years after surgery [31, 43].

Differences between sports are also reflected in the time to RTP, with recovery times typically ranging from 6–10 months. Rugby players return to play faster (9.6 months) than football players (10.6 months) [13, 31].

However, measuring and comparing RTP levels in different sports is challenging because of the lack of a standardised measurement method, highlighting the need for further research. The aim of this systematic review and meta‐analysis was to compare RTP, time to RTP, level of RTP, and rate of ACL graft failure in elite and professional athletes from different sports. It was hypothesised that the majority of athletes would RTP at their pre‐injury level.

## METHODS

A systematic search strategy was developed according to the Preferred Reporting Items for Systematic Reviews and Meta‐Analyses (PRISMA) guidelines and is registered in the PROSPERO Registry (CRD42025632248) [66, 67].

An electronic database search was performed to identify potentially relevant research articles that analysed RTP, time to RTP, level of RTP and percentage of ACL graft rupture in elite and professional athletes after ACL reconstruction. The MEDLINE (PubMed), Embase (Elsevier), and Cochrane Library databases were searched on 2 January 2025, and repeated after 2 weeks, using the following Boolean search terms: ‘ACL reconstruction’ OR ‘anterior cruciate ligament reconstruction’ OR ‘ACL’ AND ‘professional’ OR ‘elite’ OR ‘competitive’ OR ‘high‐level’ OR ‘top‐level’.

### Eligibility criteria

The literature selected for this study was selected on the basis of the following criteria.

#### Study design

Randomised controlled trials (RCTs), controlled (non‐randomised) clinical trials (CCTs), prospective and retrospective comparative cohort studies, case–control studies, and case series were included. Case reports and case series that did not report data on RTP, RTP level, time to RTP or the rate of ACL graft failure were excluded. RTP was defined as participation in at least one game in the same league as before the injury. Studies reporting on overlapping patient populations with the same outcomes were also excluded (only one such study was included in the review).

#### Participants and interventions

Studies were conducted on skeletally mature elite or professional athletes treated with ACLR and evaluated for RTP activity, time to return, level of RTP and rate of ACL graft failure. Concomitant procedures were not considered exclusionary, provided that ACLR was the primary procedure. In trials that included revision ACLR without clearly separable data, outcomes were analysed under the assumption of primary surgery.

An elite or professional athlete was defined as one who participates in national‐ or international‐level competitions in professional or amateur sports—including academy players aged 15 years or over [11]. The minimum follow‐up period included studies that analysed athletes for at least one full season after the intervention.

### Types of outcome measures

Four different outcome measures were extracted and recorded:

- Return to play: Played in at least 1 game after ACLR in the same sport activity.
- Time to return to play: The time between surgery and return to the first official game in days.
- Return to pre‐injury level: The criterion for returning to the preinjury level was playing at the same or higher level (% calculated for patients who returned to play).
- ACL graft failure: Ruptures of the ipsilateral ACL after ACLR (% calculated for patients who returned to play).

### Data collection and analysis

#### Study selection

The retrieved articles were first screened by title and, if relevant, further screened by reading the abstracts. After studies that did not meet the eligibility criteria were excluded, the entire content of the remaining articles was assessed for eligibility. To minimise the risk of bias, the authors reviewed and discussed all the selected articles, references, and articles excluded from the study. In the case of any disagreements among the reviewers, the senior investigator made the final decision. At the end of the process, further studies that might have been missed were searched manually by going through the reference lists of the included studies and relevant systematic reviews.

#### Data collection process

The data were extracted from the selected articles by the first two authors using a computerised tool created with Microsoft Access (Version 2010, Microsoft Corp, Redmond Washington). Each article was validated again by the first author before analysis. For each study, data regarding the patients were extracted (age, sex, sports practised), RTP, time to RTP, level of postoperative activity, and ACL graft failure rate.

#### Level of evidence

The Oxford Levels of Evidence set by the Oxford Centre for Evidence‐Based Medicine were used to categorise the level of evidence [52].

#### Evaluation of the quality of studies

The quality of the selected studies was evaluated using the Methodological Index for Nonrandomized Studies (MINORS) score. The checklist includes 12 items, of which the last four are specific to comparative studies. Each item was given a score of 0–2 points. The ideal score was set at 16 points for noncomparative studies and 24 points for comparative studies [76].

### Statistical analysis

We calculated the weighted average age, that is, the average age weighted according to the number of knees included in each study, and tested differences by sport with a weighted linear regression model.

Four meta‐analyses were performed on the frequency of (i) RTP, (ii) the level of return to the preinjury level, (iii) ACL graft failure and (iv) the mean time to RTP.

For the first three outcomes, a random‐effects model was performed using the DerSimonian‒Laird estimator for variance. The raw proportions were stabilised using the Freeman–Tukey double arcsine transformation. Pooled estimates were presented as pooled proportions with corresponding 95% confidence intervals (95% CIs). For the average time to RTP, we also performed a random‐effect model on raw means using the restricted maximum likelihood (REML) estimator for variance estimation. The pooled estimates were presented as pooled means with 95% CIs. In studies where the range was available instead of the standard deviation (SD), the SD was estimated on the basis of sample size and range [84]. For each outcome, between‐study variation was assessed for each model using the Cochran's Q *χ* ^2^ test for heterogeneity and the Higgins *I* ^2^ statistic. Statistical heterogeneity was defined as substantial when *I* ^2^ > 50% [35].

For subgroup analyses, we explored sport differences with univariable mixed‐effects meta‐regression models with a common between‐study variance component across subgroups [80]. As above, for the first three outcomes, we used the Der Simonian‒Laird variance estimator and the Freeman‒Tukey double arcsine outcome transformation. For the fourth outcome, the variance was estimated using the REML method. In subgroup analyses, for each outcome, heterogeneity between sports was tested with a Cochran's Q *χ* ^2^ test. For the comparison of sport categories, soccer was selected as the reference category because it had the highest frequency of studies and knees in this group.

For each model, we included sports with more than two studies with available data for each outcome. Meta‐analyses on (ii) return to the preinjury level, (iii) ACL graft failure and (iv) the average time to RTP were conducted among subjects who underwent RTP. Sensitivity analyses included all sports with at least two studies with available data for each of the first three outcomes. For time to RTP, a sensitivity test was conducted, including studies that reported only the SD in the primary study, to assess the effect of the estimated SD [85].

Publication bias and small‐study effects were assessed through funnel plots. Their asymmetry was tested with the rank correlation test and the regression test. Two‐tailed tests were performed. A *p*‐value of < 0.05 was considered statistically significant. The analysis was carried out using R (version 4.3.0, R Foundation for Statistical Computing, Vienna, Austria). URL https://www.R-project.org/), specifically with meta (version 8.0.1) and metafor packages (version 4.2.0).

## RESULTS

**Figure 1:**
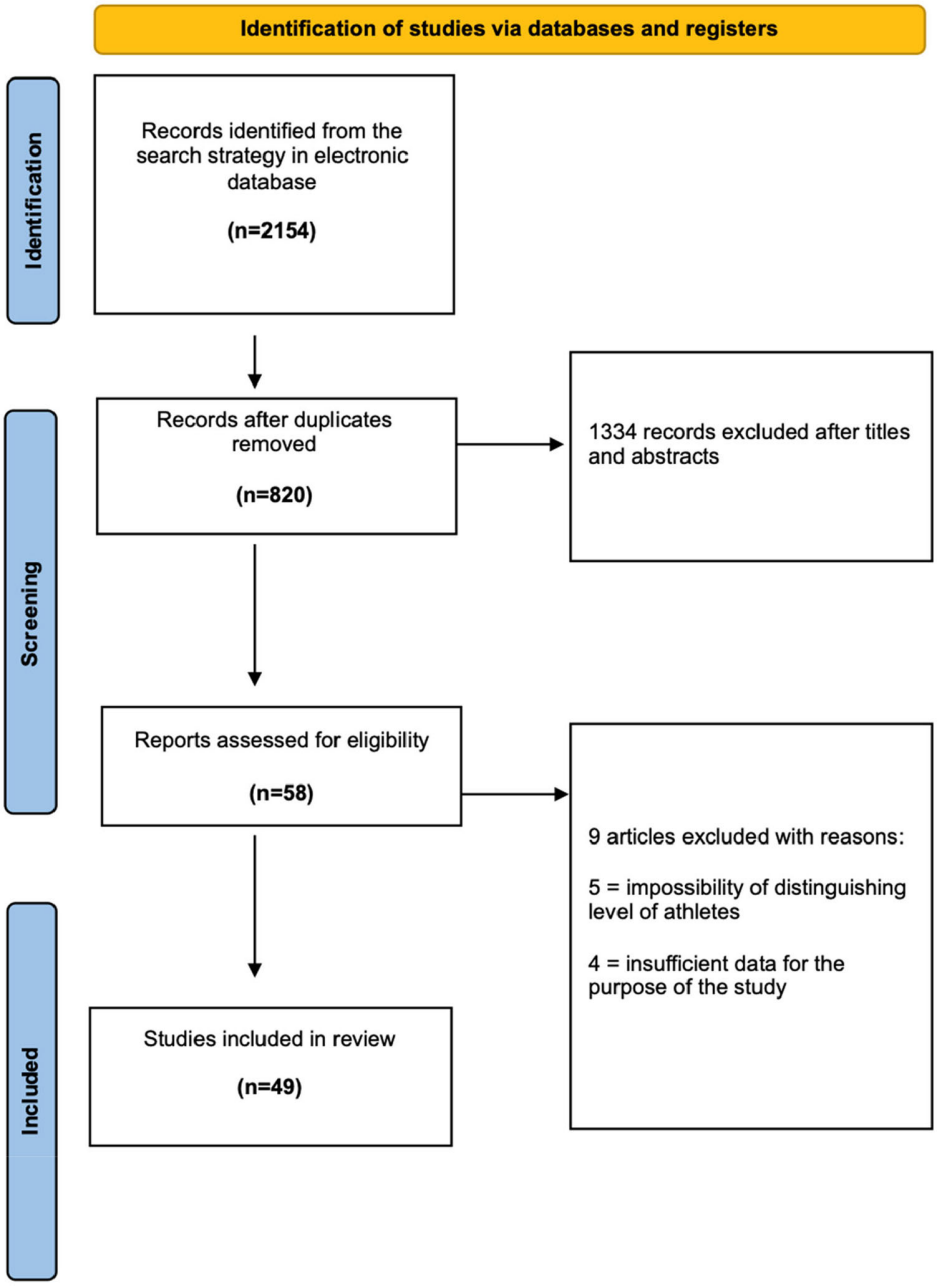
PRISMA (Preferred Reporting Items for Systematic Reviews and Meta‐Analyses) flow chart indicating research article inclusion for final analysis.

Initially, a thorough search of the three electronic databases yielded 2154 records. The titles and abstracts of 820 studies were reviewed after 1334 duplicates were eliminated. After reviewing the title and abstract, 762 trials were removed and 58 full‐text articles were assessed for eligibility. Finally, the reviewers excluded nine studies after evaluating the full texts, leaving 49 articles included in the final analysis of this review. The PRISMA diagram is shown in Figure 1.

### Study characteristics

All patients and study characteristics are reported in Table 1. A total of 11 sports and 4463 knees were included.

**Table 1:** Analysis of each study included in the meta‐analysis by follow‐up time, number of athletes, gender and return to sport criteria.

Authors	Level of evidence	MINORS	Analysis period	Definition of return to play	Definition of same level of return	Number of patients	Gender (M/F)	Age (range) ± SD, [IQR] (years)
Australian Football League								
Liptak and Angel [50]	IV	12	3 seasons postop	Playing at least 1 further senior AFL match after undergoing an ACLR	The criterion for return to preinjury level was that the players' number of disposals postinjury was equal to or greater than the number of exposures before their injury	115	115/0	24 (17‐35)
Lai et al. [47]	IV	13	14‐year time span	Playing at least 1 further senior AFL match after undergoing an ACLR	The criterion for return to preinjury level was the number of AFL home and away matches missed and the number of days between ACL injury and return to play	158	158/0	23.6
Lai et al. [48]	IV	11	14‐year time span	Playing at least 1 further senior AFL match after undergoing an ACL reconstruction	The criterion for return to preinjury level was the number of AFL home and away matches missed and the number of days between ACL injury and return to play	104	104/0	n.a.
Badminton								
Tan et al. [79]	III	14	Athletes who sustained an ACL injury between 2001 and 2021	Having completed a match on the Badminton World Federation tour in a competition of a similar level compared to preinjury	Having a Badminton World Federation world ranking within 10 spots or higher after the ACL injury compared to the world ranking at the time of injury	66	26/40	23.2 ± 3.7
Baseball								
Erickson et al. [25]	III	14	Players underwent ACLR between 2010 and 2015	Played in at least 1 game after ACLR	The criterion for return to preinjury level was based on in‐game performance variables analysed as an average over the pre‐ACLR and post‐ACLR course of the player's career	124	124/0	23.7 ± 4.1
Mai et al. [53]	III	13	5.6 years postop	Being on the active roster for at least 1 regular or postseason game after treatment	Player performance was measured based on the role of either a pitcher or hitter	21	21/0	30.0
Basketball								
Namdari et al. [61]	III	13	2 seasons postop	Playing at least 1 further senior WNBA match after undergoing an ACL reconstruction	The criterion for return to preinjury level was based on games played, field goal percentage, average minutes played, points, assists, rebounds, steals, and blocks per game	18	0/18	26.8 (21.7–31.3)
DeFroda et al. [23]	IV	11	2 seasons postop	The first game on an active roster defined successful return to play	The criterion for return to preinjury level was based on games started, games played, minutes played, and player efficiency rating were recorded for seasons before and after surgery	26	26/0	25.3 (19–38)
Abbas et al. [1]	III	12	7 seasons postop	The first game on an active roster defined successful return to play	The criterion for return to preinjury level was based on player efficiency rating, contract length and salary information	54	54/0	24.9 ± 3.93
Busfield et al. [17]	IV	12	4 seasons post‐op	The first game on an active roster defined successful return to play	The criterion for return to preinjury level was based on player efficiency rating	27	27/0	25.7
Mai et al. [53]	III	13	4.8 years postop	Being on the active roster for at least 1 regular or postseason game after treatment	The criterion for return to preinjury level was based on performance was measured using the player efficiency rating	76	76/0	26.2
Kester et al. [41]	IV	12	14‐year time span	Being on the active roster for at least 1 regular or postseason game after treatment	The criterion for return to preinjury level was based on player efficiency rating	79	79/0	n.a.
Harris et al. [34]	IV	12	37‐year time span	A player was deemed to have RTP in the NBA if he played in any NBA game after surgery	The criterion for return to preinjury level was based on return at the same or higher level than before surgery	55	55/0	25.7 ± 3.5
Nwachukwu et al. [64]	IV	11	2 seasons postop	Return to play status was assessed based on whether the player returned to basketball participation at the prior professional level	Return to play performance was assessed by comparing pre‐injury functional performance with functional performance in RTP Season 1 and RTP Season 2	12	12/0	24.6 ± 4.4 (19–33)
Football								
Daruwalla et al. [22]	IV	10	ACL reconstruction from 2004 to 2010	Achieving full, unrestricted participation in a full‐contact practice, scrimmage, or regular season game at any time after the date of surgery	The criterion for return to preinjury level was based on return at the same or higher level before surgery	184	184/0	n.a.
Cinque et al. [20]	III	12	1 season postop	A player was deemed to have RTP if he played in at least 1 NFL game after ACL reconstruction	return to preinjury level was based on return at the same or higher level before surgery	73	73/0	26.3 ± 2.6
Manoharan et al. [55]	IV	13	2 season postop	For each player, it was determined if the player was successful in RTP after ACLR, where RTP was defined as playing in at least 1 NFL game after surgery	The criterion for return to preinjury level was based on Performance statistics were gathered for each player, including career games played, percentage of games started, age at time of injury, and timing of injury	76	76/0	25.8
Mody et al. [59]	IV	13	3 seasons postop	Return to play was defined as playing in at least 1 snap in at least 1 regular season NFL game after ACL injury	The criterion for return to preinjury level was based on snap count, games played, games started, and approximate value	312	312/0	30.65
Carey et al. [19]	IV	14	3 seasons postop	Return to competition was defined as participation in an American NFL game in the regular season for at least 1 play	The criterion for return to preinjury level was based on games played, total (rushing and receiving) yards, and touchdowns. and power rating	31 (33 knees)	31/0	27.1 ± 0.6
Erickson et al. [27]	IV	14	4.8 years	A player was deemed to RTS if he played in any NFL game after surgery	The criterion for return to preinjury level was based on average in‐game performance	13 (14 knees)	13/0	27.2 ± 2.39
Longstaffe et al. [51]	IV	14	ACL reconstruction from 2002 through 2017	Successful RTP was defined as returning to play in an actual game, either preseason or regular season	The criterion for return to preinjury level was based on playing at the same or higher level	42 (43 knees)	42/0	26.1 ± 2.6
Read et al. 2017 [71]	III	13	3 seasons postop	Successful RTP was defined as returning to play in an actual game, either preseason or regular season	The criterion for return to preinjury level was based on Yearly performance statistics	38	38/0	27.6 ± 3.1
Shah et al. [74]	IV	11	2 seasons postop	Successful RTP was defined as actual game play in regular season NFL football games after ACL reconstruction	The criterion for return to preinjury level was based on playing at the same or higher level	49	49/0	n.a.
Mai et al. [53]	III	11	3.5 years postop	Being on the active roster for at least 1 regular or postseason game after treatment	The criterion for return to preinjury level was based on standardised, previously published scoring system based on pertinent statistics important to the individual player's position	205	205/0	26.6
Khair et al. [42]	IV	11	27‐year time span	Return to game play was defined as returning to play in a regular‐season game	Successful return to previous participation was defined as return to a level of participation equal to the level the player had reached before injury	43	43/0	n.a.
Eisenstein et al. [24]	IV	13	ACL reconstruction from 2013 to 2015	Return to play was defined as playing at least 1 down in a regular‐season NFL football game	Successful return to previous participation was defined as return to a level of participation equal to the level the player had reached before injury	92	92/0	25.96
Hockey								
Erickson et al. [26]	III	13	4 years after surgery	A player was deemed to have RTP if he played in any NHL game after surgery or if he is less than 12 months out from date of injury but is expected to RTP	The criterion for return to preinjury level was based on average in‐game performance data per season	36 (37 knees)	36/0	27.1 ± 4.05
Mai et al. [53]	III	12	2–3 seasons postop	Being on the active roster for at least 1 regular or postseason game after treatment	The criterion for return to preinjury level was based on performance score based on a previously published scoring system reported in the literature to assess performance after orthopaedic procedures	48	48/0	28.1
Kabbadi								
Arora et al. [4]	IV	11	1 year	Return to sport was assessed using a subjective patient centric questionnaire	Return to sport and was assessed using a subjective patient centric questionnaire	93	78/15	22.4 ± 2.7
Rugby								
Hurley et al. [38]	IV	13	2 years	Successful RTP was defined as returning to play in an actual game, either preseason or regular season	The criterion for return to preinjury level was based on playing at the same or higher level	15	15/0	22.3 ± 5.2
Takazawa et al. [78]	IV	14	11 years	All players completed a study‐specific questionnaire involving a series of structured questions regarding their participation in rugby at any time after their surgery	All players completed a study‐specific questionnaire involving a series of structured questions regarding their participation in rugby at any time after their surgery	12	12/0	26.0 ± 2.2
Jones et al. [39]	IV	12	2 years	RTP was defined as participation in a professional match or in national/international‐level competition	The criterion for return to preinjury level was based on the Tegner score as moving from the top division in rugby results in a drop in the Tegner score	125	114/11	23.4 ± 4.0
Soccer								
Zaffagnini et al. [87]	IV	13	4 years	Successful RTP was defined as returning to play in an actual game, either preseason or regular season	The criterion for return to preinjury level was based on playing at the same or higher level	21	21/0	22.9 ± 5.4
Barth et al. [7]	IV	12	3 seasons postop	successful RTP was defined as an athlete who played in a minimum of one professional game in any league following the injury	The criterion for return to preinjury level was based on specific player performance statistics	176	176/0	26.1 ± 3.8
Arundale et al. [5]	III	10	2 years	Successful RTP was defined as returning to play in an actual game, either preseason or regular season	The criterion for return to preinjury level was based on playing at the same or higher level	54	54/0	n.a.
Forsythe et al. [29]	III	13	4 years	Successful RTP was defined as returning to play in an actual game, either preseason or regular season	The criterion for return to preinjury level was based on change in performance metrics	51	51/0	24.9 ± 4.1
Mazza et al. [58]	IV	13	3 seasons postop	The percentage of players, among all the injured players, having played at least 1 game at a professional level after ACLR	The criterion for return to preinjury level was based on playing at the same or higher level	183	183/0	25.4 ± 3.9 (18–37)
Farinelli et al. [28]	IV	13	3 seasons postop	Successful RTP was defined as returning to play in an actual game, either preseason or regular season	The criterion for return to preinjury level was based on playing at the same or higher level	27	27/0	23.15 ± 4.3 (18–34)
Abed et al. [2]	IV	12	2 years after return to sport	RTP was defined as any player who played at least 1 min in at least 1 NWSL game after the injury	The criterion for return to preinjury level was based on FBref database	30	0/30	24.8 (22.5–28)
Szymski et al. [77]	IV	12	3 seasons postop	Successful RTP was defined as returning to play in an actual game, either preseason or regular season	The criterion for return to preinjury level was based on playing at the same or higher level	120	120/0	24.7 ± 4.3 (18–32)
Waldén et al. [83]	IV	13	3 years	RTP was defined as the number of days from injury or reconstruction to full training with the team without restrictions (return to training) and to the first match appearance with the first team, reserve team, under‐21 team or a national team (return to match play)	Return to the same level of play was defined as return to the highest national league level, irrespective of country, and to lower level of play as all levels below the highest national league	134	134/0	24.7 ± 4.5
Borque et al. [12]	III	14	10 years	Successful RTP was defined as returning to play in an actual game, either preseason or regular season	The criterion for return to preinjury level was based on playing at the same or higher level	82	82/0	25.2 ± 4
Krutsch et al. [45]	IV	11	9 seasons postop	Successful RTP was defined as returning to play in an actual game, either preseason or regular season	The criterion for return to preinjury level was based on playing at the same or higher level	63	63/0	24.8 ± 3.8
Jones et al. [39]	IV	13	2 years	RTP was defined as participation in a professional match or in national/international‐level competition	The criterion for return to preinjury level was based on the Tegner score as moving from the top division in rugby results in a drop in the Tegner score	234	234/0	23.3 ± 4.3
Waldén et al. [82]	IV	11	Seasons from 2001 to 2009	RTP was defined as the number of days from injury or reconstruction to full training with the team without restrictions (return to training) and to the first match appearance with the first team, reserve team, under‐21 team or a national team (return to match play)	Return to the same level of play was defined as return to the highest national league level, irrespective of country, and to lower level of play as all levels below the highest national league	71	n.a.	24.3 ± 4.5
Balendra et al. [6]	IV	12	2 years after postop	Playing in at least one competitive football match at a professional level following ACLR	The criterion for return to preinjury level was based on the Tegner activity scale	215 (232 knees)	205/17	23.3 ± 4.4
Pinheiro et al. [68]	IV	13	5 years postop	Playing in at least one competitive football match at a professional level following ACLR	The criterion for return to preinjury level was based on the Tegner activity scale	200	200/0	24.1 ± 4.2
Bonanzinga et al. [10]	IV	13	8 seasons postop	Playing in at least one competitive football match at a professional level following ACLR	The criterion for return to preinjury level was based on the Tegner activity scale	28	28/0	25.3 ± 5.0
Niederer et al. [62]	IV	14	5 years postop	Return to play was defined as (a) having played at least one game at a competitive level (irrespective of the minutes on field) after the reconstruction (or after the matching point of time); and (b) for the long‐term sustainability rates: not having retired from playing competitive football	Pre‐injury level was calculated considering the countries' ranking within the UEFA country coefficient ranking and the respective league (1st or 2nd) for the players who changed the team (or played for the reserve team). If the player stayed in the same team and the team advances or relegates to another league, pre‐injury level was calculated accordingly	125	125/0	25.3 ± 4.2 (18–37)
Ghali et al. [30]	IV	12	3 years postop	Date of return is defined as the first game back in competition (not training)	The criterion for return to preinjury level was based on matches played, minutes played, goals, assists, shots, shots on target, tackles attempted, tackles won percentage, pass completion percentage, penalty kicks, interceptions, blocks, and clearances	23	23/0	25.3 ± 3.40
Howard et al. [37]	IV	12	8‐years span time	Successful RTP was defined as returning to play in an actual game, either preseason or regular season	The criterion for return to preinjury level was based on playing at the same or higher level	78	0/78	19.3 (17–22)
Ski Jumping								
Oronowicz et al. [65]	IV	13	3 seasons postop	Successful RTP was defined as returning to ski international competition	Based on the outcome, athletes' performance was classified as better, similar or inferior, comparing post‐ to preinjury results.	18	11/7	23.7
Wrestling								
Marigi et al. [57]	IV	14	5.9 years	Playing in at least one wrestling match following ACLR	The criterion for return to preinjury level was based on the Tegner activity scale	107	106/1	17 (15–18)

Abbreviations: ACL, anterior cruciate ligament; ACLR, anterior cruciate ligament reconstruction; AFL, Australian Football League; F, female; IQR, interquartile range; M, male; n.a., not available; NBA, National Basketball Assocation; NFL, National Fooball League; NHL, National Hockey League; NWSL, National Women's Soccer League; postop, postoperative; RTP, return to play; SD, standard deviation; UEFA, Union of European Football Associations; WNBA, Women's National Basketball Association;.

### Age

The mean age of athletes at the time of surgery was significantly higher for football and hockey players (*p* < 0.05). The detailed results are reported in Table 2.

**Table 2:** Characteristics of studies by sport.

	*N* of studies	*N* of knees	Weighted mean age in years^a^	
	*N* = 44	*N* = 3528		
	*n* (%)	*n* (%)	Mean ± SD	*p* value
Australian Football League	2 (4.5)	273 (7.7)	23.8 ± 0.2	0.48
Badminton	1 (2.3)	66 (1.9)	23.2 ± 0.0	0.46
Baseball	2 (4.5)	145 (4.1)	24.6 ± 2.2	0.78
Basketball	7 (15.9)	271 (7.7)	25.7 ± 0.6	0.09
Football	6 (13.6)	468 (13.3)	26.4 ± 0.5	0.002*
Hockey	2 (4.5)	85 (2.4)	27.7 ± 0.5	0.02*
Kabbadi	1 (2.3)	93 (2.6)	22.4 ± 0.0	0.14
Rugby	3 (6.8)	152 (4.3)	23.5 ± 0.8	0.42
Ski Jumping	1 (2.3)	18 (0.5)	23.7 ± 0.0	0.83
Soccer	18 (40.9)	1877 (53.2)	24.3 ± 1.4	Reference category
Wrestling	1 (2.3)	80 (2.3)	17.0 ± 0.0	<0.001*

^a^ Standard deviation (SD) calculated on the weighted mean age;

* Statistical significant difference.

### Return to play

**Figure 2:**
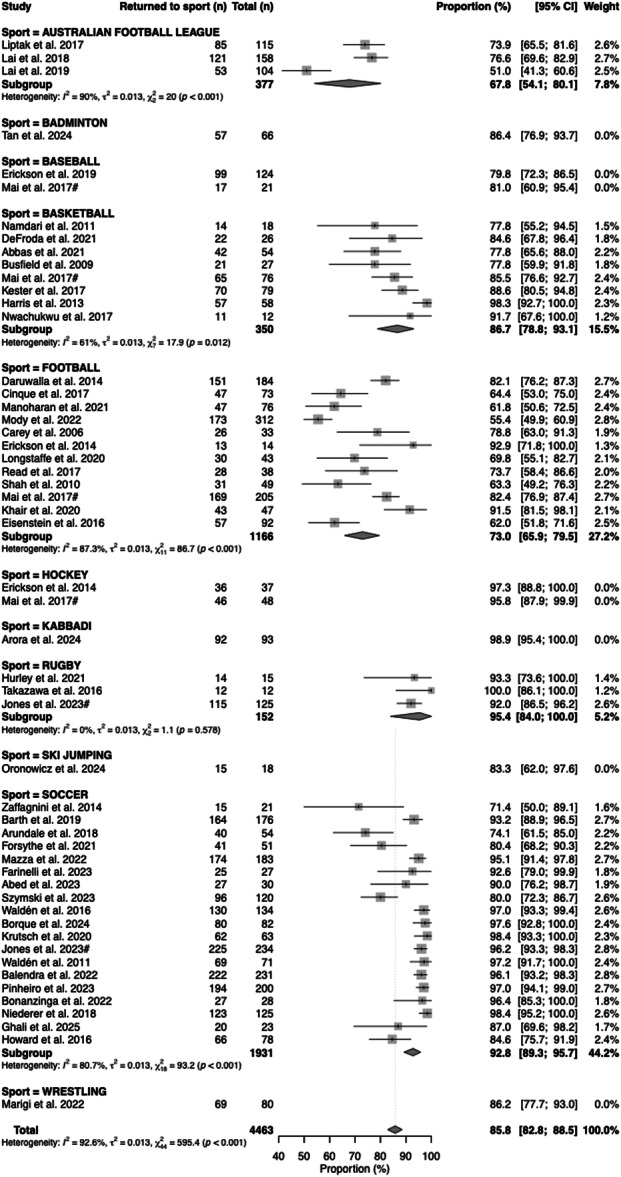
Forest plot of RTP among different sports after ACL reconstruction. ACL, anterior cruciate ligament; CI, confidence interval; RTP, return to play.

The pooled meta‐analysis revealed that 85.8% (95% CI 82.8–88.5) of athletes RTP. A lower rate of RTP was found in Australian football (67.8% [95% CI 54.1–80.1]; *p* < 0.001) and football (73.0% [95% CI 65.9–79.5]; *p* < 0.001) than in the reference group (soccer − 92.8% [95% CI 89.3–95.7]). The forest plot in Figure 2 shows in detail the RTP for each study and sport.

### Level of RTP

**Figure 3:**
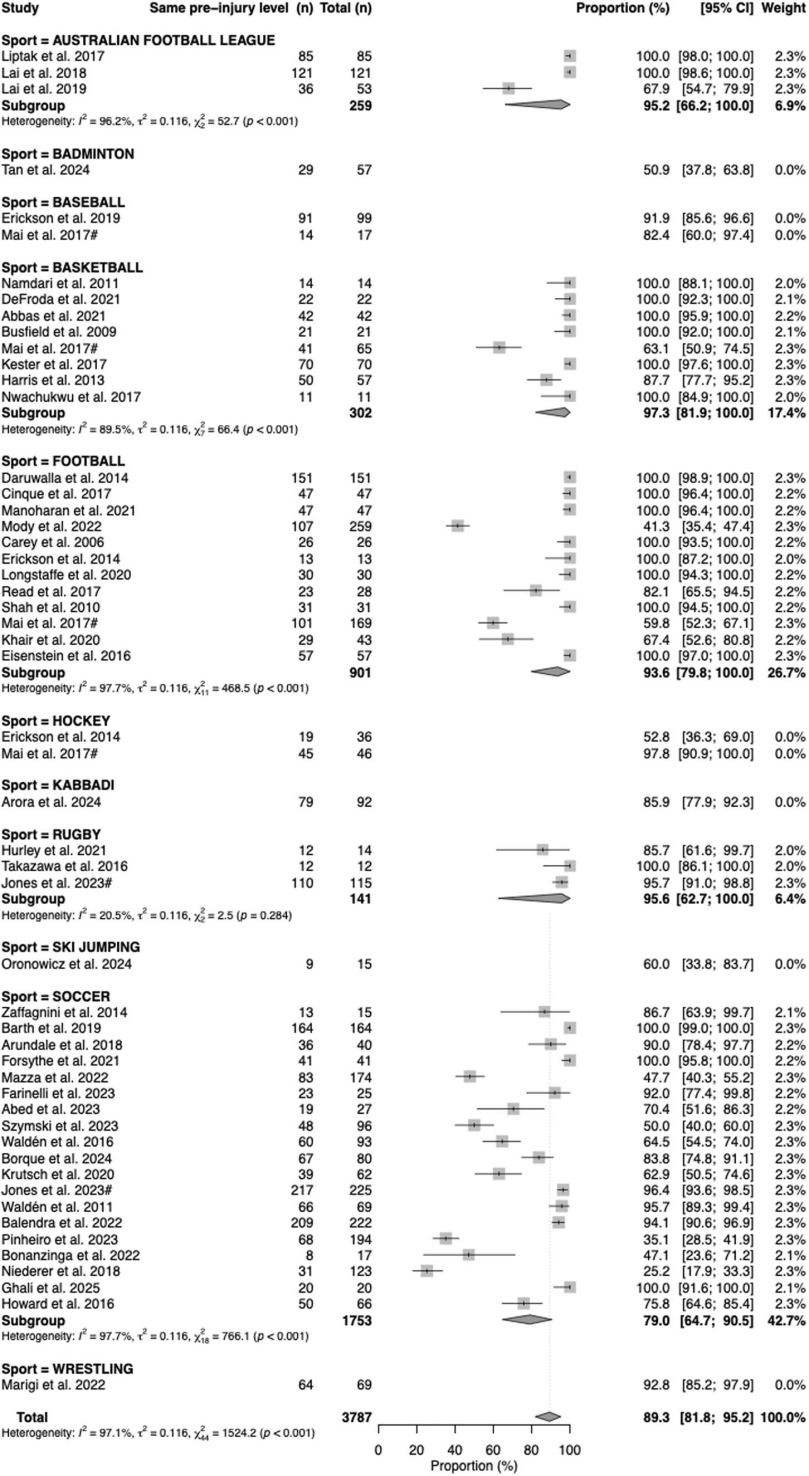
Forest plot of the level of RTP among different sports after ACL reconstruction. ACL, anterior cruciate ligament; CI, confidence interval; RTP, return to play.

The pooled meta‐analysis revealed that 89.3% (95% CI 81.8–95.2) of athletes returned to their preinjury level. The meta‐analysis revealed no difference (*p* > 0.05) in the RTP rates between studies, ranging from 79.0% (soccer) to 97.3% (basketball). The forest plot in Figure 3 shows the RTP for each study and sport in detail.

### Time to RTP

**Figure 4:**
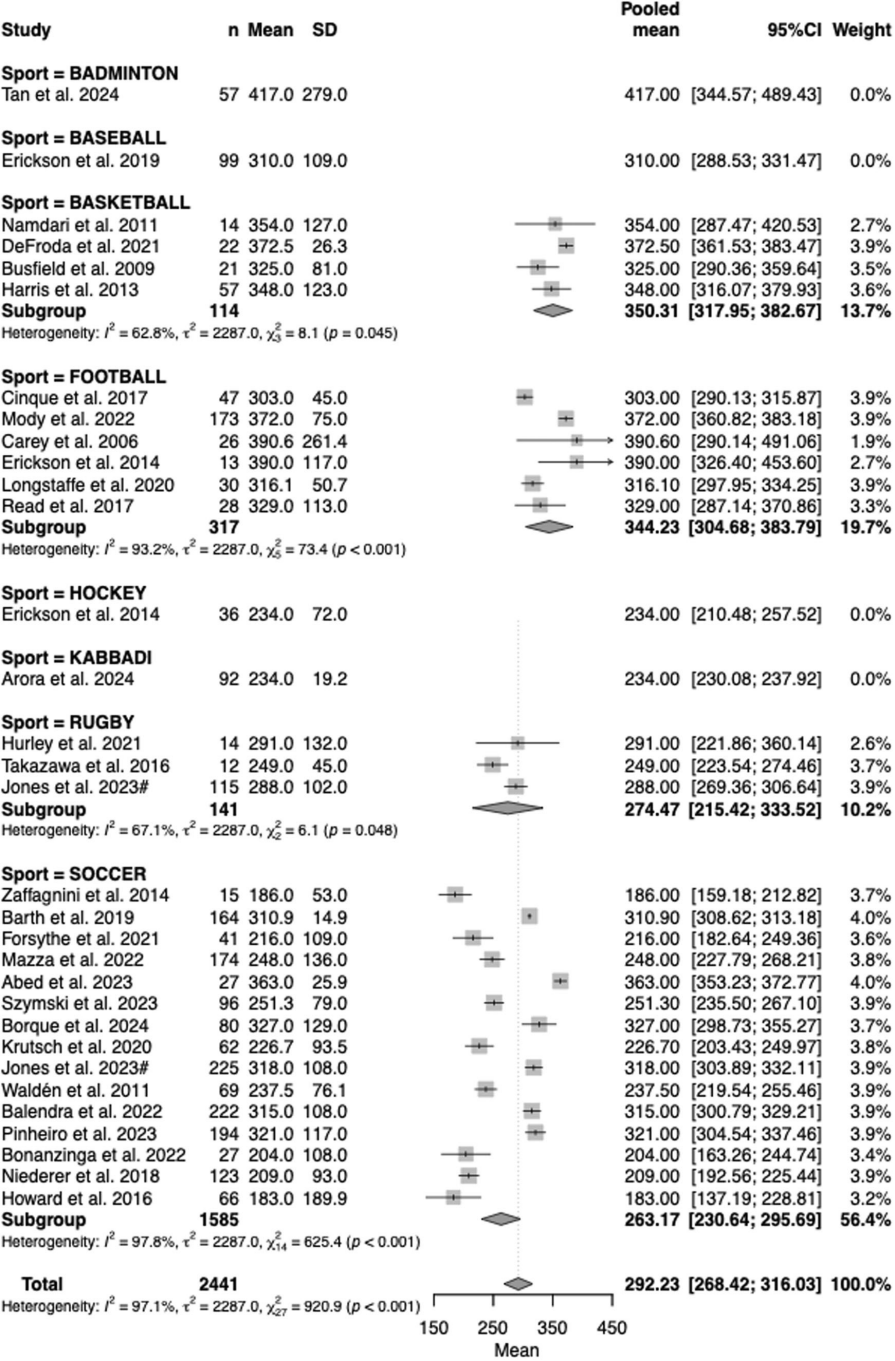
Forest plot of time to RTP among different sports after ACL reconstruction. ACL, anterior cruciate ligament; CI, confidence interval; RTP, return to play; SD, standard deviation.

The mean time to RTP was estimated to be 292 days (95% CI 268–316). Soccer players had a faster RTP (263 days [95% CI 231–296]) than basketball (350 days [95% CI 318–383]; *p* = 0.0047) and football (344 days [95% CI 305–384]; *p* = 0.006) players. The forest plot in Figure 4 shows the RTP for each study and sport.

### Graft failure rate

**Figure 5:**
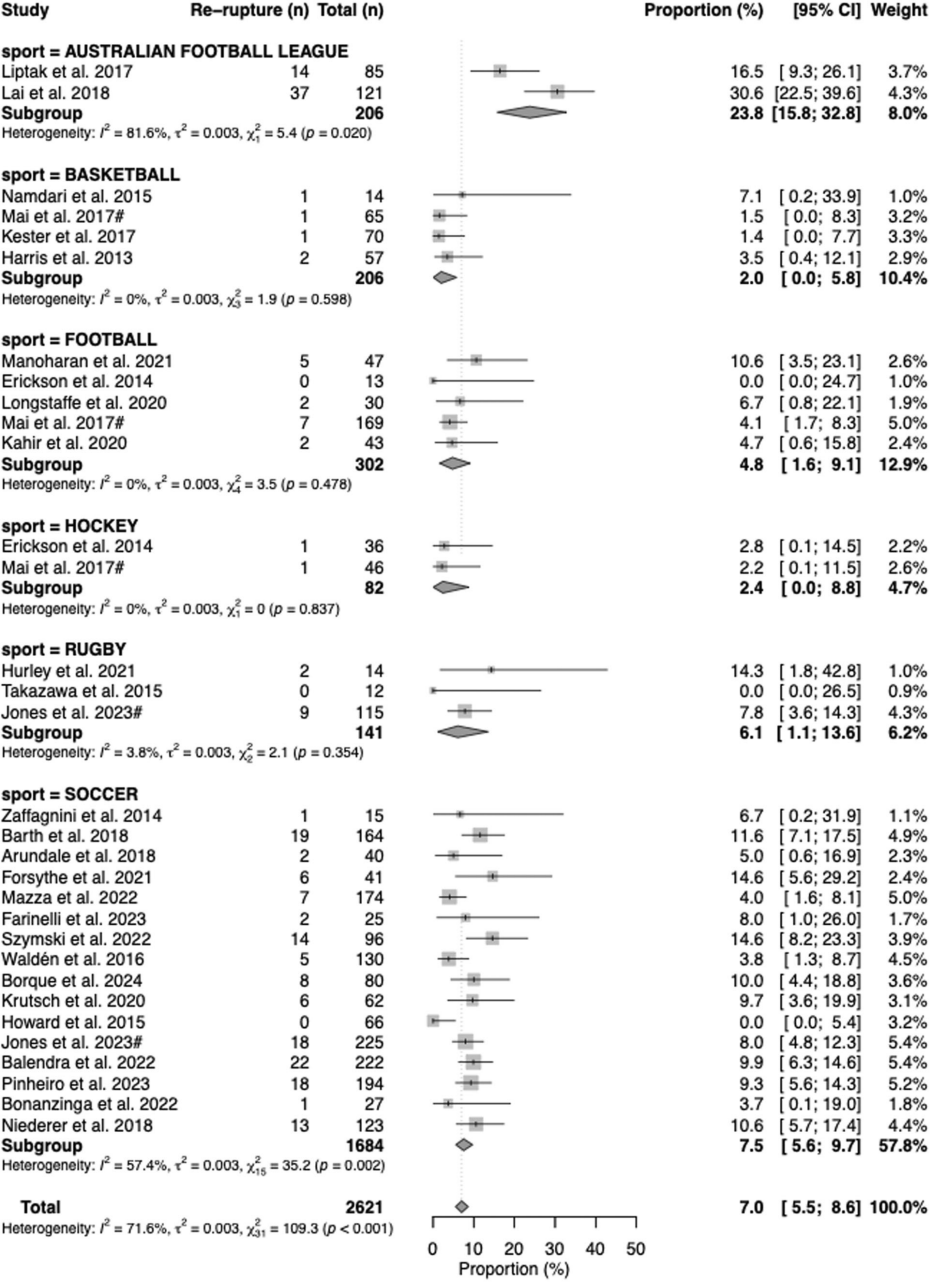
Forest plot of the failure rates among different sports after ACL reconstruction. ACL, anterior cruciate ligament; CI, confidence interval.

The pooled ACL graft failure rate was estimated to be 7.0% (95% CI 5.5–8.6). Australian football (23.8% [95% CI 15.8–32.8]; *p* < 0.001) reported a higher failure rate than the reference group (soccer 7.5% [95% CI 5.6–9.7]). Basketball reported a lower failure rate (2.0% [95% CI 0.0–5.8]; *p* = 0.042). The forest plot in Figure 5 shows in detail the failure rates for each study and sport.

## DISCUSSION

The most important finding of the present meta‐analysis was that more than 85% of elite and professional athletes returned to sport after ACL reconstruction, and almost 90% returned to their preinjury level, with an ACL graft failure rate of 7.0% and a mean return to sport at 292 days. Australian Football athletes and football athletes have a lower return to sport rate, whereas basketball and football players have a slower return to sport. Basketball players have a lower reinjury rate than Australian football players.

Few studies in the literature have analysed and compared the return to sport among elite and professional athletes and reported results similar to ours. In 2018, Lai et al. determined the rate of return to the preinjury level of sport after ACL reconstruction in elite athletes. The pooled RTP rate was 83%. The mean time to RTP ranged from 6 to 13 months. The pooled graft failure rate was 5.2%. Six out of nine studies that included an uninjured control group reported no significant deterioration in athletic performance following ACL reconstruction [46].

A lower rate of return to sport was reported by Ardern et al., who revealed that, on average, 81% returned to any sport, 65% returned to their preinjury level of sport, and 55% returned to a competitive level of sport after surgery. The same author highlighted similar results in a previous study analysing athletes after ACL reconstruction: 82% of participants had returned to some type of sport, 63% had returned to their preinjury level of participation, and 44% had returned to competitive sports at the final follow‐up [3].

In 2020, Ross et al. analysed the impact that an ACL injury has on the ability to RTP and the post‐ACLR performance level in American football players. The rate of RTP after ACLR in football players was 67.2%, and the mean time to return was 11.6 months. Although there was considerable heterogeneity in the study designs and outcomes measured, in general, the majority of football players experienced a greater decline from their preinjury level of performance than did the controls over the same period [72].

In soccer players, Manojlovic et al. assessed RTP and performance after ACLR. They reported an RTP rate of 72% and a return to preinjury sports level of 53% after ACLR. In addition, recent evidence provided in this literature review demonstrated that the mean time to RTP was 264 days or 8.7 months. The average career length of soccer players after ACL surgery is approximately 4–5 years [56].

It is imperative to ascertain the functional status of athletes prior to their return to sport to guarantee the most favourable results, as demonstrated by prior research. However, there is no consensus on the most effective method for determining an athlete's RTP status after ACLR. According to a recent systematic review, the most commonly used criteria for RTP are subjective knee stability, degree of laxity on physical examination, and time since surgery [13, 16].

Another outcome of our study was the time to return to sport, with variable results across different disciplines. Time was the most commonly used criterion to clear patients for RTS after ACLR. The time following ACL reconstruction is crucial for the recovery of physical function and the 'ligamentisation' of the graft. Studies suggest that the rupture rate can be reduced by 50% by delaying RTS beyond 9 months following ACL reconstruction [8, 21].

Unfortunately, in the literature, the description of return to sport guidelines is infrequently and variably reported. There are no definitive rules for allowing a safe return to unrestricted sports, despite the abundance of peer‐reviewed evidence available in the medical literature. Following ACL surgery, return to sport depends on a number of patient‐, knee‐, and ligament‐specific factors. Clinical investigations often report subjective and clinical outcome scores that are validated, responsive, and trustworthy. The preinjury competitive level played, the patient's postinjury ambitions, and the level of sport attained after ACL surgery all influence how broadly and differently the capacity to return to sport is characterised [33, 70].

The results of this systematic review reported a ACL graft failure rate of 7%; it is estimated that the incidence of reconstruction after ACLR is between 1.7% and 7.7%. A 25‐year follow‐up study revealed that the graft failure rate after ACLR was approximately 9%. Although the risk of revision or graft rupture after ACLR is not high, secondary ACLR is more technically demanding than primary ACLR, with a higher failure rate and lower patient satisfaction, which has a significant impact on the likelihood of returning to sport [73, 88].

An athlete's ability to return to sport may also be influenced by psychological factors. Depression, self‐efficacy, and kinesiophobia (fear of reinjury or movement) can all affect athletes during the rehabilitation process. In fact, clinically diagnosed depression is one of the most debilitating psychological factors affecting clinical outcomes after ACLR [69]. This condition has been identified preoperatively in nearly 40% of ACLR candidates [69]. Therefore, it is prudent to identify and address psychological factors during the recovery process to better equip athletes for their return to competition. Interventions such as guided imagery, relaxation, behavioural counselling, goal setting, and coping modelling have yielded inconsistent results; however, they appear to be generally promising [69].

### Limitations

The overall return to sport rate of 85% should be interpreted with some caution. There was considerable statistical heterogeneity among the studies, which may be explained by several factors. The included studies employed different methods to identify and recruit elite and professional athletes, used varying criteria to assess return to sport, and the rate of return may have been influenced by the type of sport played. The inconsistent lengths of follow‐up between studies may have affected the calculations of pooled return to sport and ACL graft failure rates. Furthermore, graft selection and time to ACL graft failure were not evaluated, and concomitant surgery was not considered an exclusion criterion. In most of the studies included in the meta‐analysis, data from patients who had concomitant surgeries could not be extrapolated, or patients who underwent ACL revision surgery were considered on par with those who had undergone ACLR for the first time. Finally, this meta‐analysis did not consider contralateral versus operated ACL failure rates.

Therefore, it is important that future studies specify the length of follow‐up, the graft used, and concomitant surgery performed.

## CONCLUSIONS

Following ACLR, more than 85% of elite and professional athletes returned to play and almost 90% returned to their preinjury level, with a ACL graft failure rate of 7.0% and a mean return to play at 292 days. Athletes and their treating physicians can utilise these findings to set reasonable expectations for their return to competition after ACLR.

## AUTHOR CONTRIBUTIONS

**Riccardo D'Ambrosi**: Conception; design; writer; materials; data collection; literature review. **Andrea Marchetti**: Design; data collection; processing; analysis; writer; materials; literature review. **Luca Farinelli**: Conception; design; materials; literature review. **Amit Meena**: Literature review; materials; design; conception; processing. **Piero Franco**: Writing; analysis literature review; analysis; data collection. **Luca Maria Sconfienza**: Conception; supervision; interpretation; critical review. **Riccardo Cristiani**: Conception; critical review; materials; data collection; processing; interpretation. **Elmar Herbst**: Writer; critical review; literature review; processing; interpretation; supervision. **Cristoph Kittl**: Conception; writer; critical review; literature review; processing; interpretation. **Mirco Herbort**: Supervision; literature review; materials; writer; critical review. **Elisabeth Abermann**: Literature review; materials; design; conception; analysis. **Christian Fink**: Conception; supervision; interpretation; critical review.

## CONFLICT OF INTEREST STATEMENT

The authors declare no conflicts of interest.

## ETHICS STATEMENT

The authors have nothing to report.

## Data Availability

Raw data are available upon request to the corresponding author.
